# Cannabis use and nonuse in patients with first-episode psychosis: A systematic review and meta-analysis of studies comparing neurocognitive functioning

**DOI:** 10.1192/j.eurpsy.2019.9

**Published:** 2020-01-31

**Authors:** Teresa Sánchez-Gutiérrez, Belén Fernandez-Castilla, Sara Barbeito, Ana González-Pinto, Juan Antonio Becerra-García, Ana Calvo

**Affiliations:** 1 Faculty of Health Science, Universidad Internacional de La Rioja (UNIR), Madrid, Spain; 2 Faculty of Psychology and Educational Sciences, KU Leuven, University of Leuven, Leuven. Belgium; 3 Hospital Universitario de Alava, Servicio de Psiquiatría, BIOARABA, CIBERSAM, Universidad del País Vasco, Leioa, Spain

**Keywords:** Cannabis, first episode, marihuana, neurocognition, psychosis

## Abstract

**Background.:**

The implications of cannabis use in the onset of early psychosis and the severity of psychotic symptoms have resulted in a proliferation of studies on this issue. However, few have examined the effects of cannabis use on the cognitive symptoms of psychosis (i.e., neurocognitive functioning) in patients with first-episode psychosis (FEP). This systematic review and meta-analysis aim to assess the neurocognitive functioning of cannabis users (CU) and nonusers (NU) with FEP.

**Methods.:**

Of the 110 studies identified through the systematic review of 6 databases, 7 met the inclusion criteria, resulting in 14 independent samples and 78 effect sizes. The total sample included 304 CU with FEP and 369 NU with FEP. The moderator variables were age at first use, duration of use, percentage of males, and age.

**Results.:**

Effect sizes were not significantly different from zero in any neurocognitive domain when users and NU were compared. Part of the variability in effect sizes was explained by the inclusion of the following moderator variables: (1) frequency of cannabis use (*β* = 0.013, *F* = 7.56, *p* = 0.017); (2) first-generation antipsychotics (*β* = 0.019, *F* = 34.46, *p* ≤ 0.001); and (3) country where the study was carried out (*β* = 0.266, *t* = 2.06, *p* = 0.043).

**Conclusions.:**

This meta-analysis indicates that cannabis use is not generally associated with neurocognitive functioning in patients with FEP. However, it highlights the deleterious effect of low doses of cannabis in some patients. It also stresses the importance of the type of antipsychotic prescription and cannabis dose as moderator variables in the neurocognitive functioning of CU with FEP.

## Introduction

In most countries, cannabis is categorized as a drug of abuse, and its recreational use is strictly prohibited [1], while in others it is perceived as a benign, relatively harmless substance [2,3]. This antagonism could be explained in part by differences in the percentage of cannabis compounds (mainly, tetrahydrocannabinol [THC] and cannabidiol [CBD]), which have geographical implications. Traditional production of cannabis resin (*hashish*), which remains prevalent in the Rif (North Africa) [4], could generate a 60% more potent form of cannabis [5]. However, new extraction techniques are now being used, mainly in central Europe and the United Kingdom, to produce extremely high-potency concentrates containing up to 75% THC. These include skunk, butane hash oil, and “Spice” [6,7].

Cannabis use may be considered a stressor for genetically vulnerable individuals [8–10]. Previous studies have reported that cannabis use increases the likelihood of early onset psychosis in risky individuals [11–15], and it could worsen the severity of psychotic symptoms [16–18]. Previous systematic reviews reported a twofold greater risk of developing a psychotic disorder in cannabis users (CU) than in nonusers (NU) [19–21]. In addition, cannabis use may induce acute cognitive effects that could diminish with abstinence [22]. Studies about the biological basis of the effects of cannabis in the general population report that smoking cannabis dysregulates the endocannabinoid system, which restores homeostasis in cases of severe brain damage, such as inflammatory processes and neurocognitive impairment [23]. In patients with first-episode psychosis (FEP), the various anomalies of the endogenous cannabinoid system include potentially increased levels of cannabinoids in the frontal cortex and cerebrospinal fluid even before the use of cannabis [24,25]. Hence, changes to this atypical endocannabinoid system caused by external cannabinoid intake have been reported to be one of the main risk factors for the onset of FEP [26]. A dose–response relationship has been established for this risk-factor, namely, the higher the levels of cannabis exposure, the higher the risk for onset of psychosis [2,27,28]. Moreover, cannabis use may reduce the time to onset of psychosis [29–31] and may affect neurocognitive functioning [32]. The relationship between cannabis use and neurocognitive functioning in FEP is supported by a considerable body of empirical evidence. However, findings on the extent of this relationship are unclear. Several studies suggest a decrease in the executive function, verbal memory and working memory of CU with FEP [33,34], whereas other studies, including one meta-analysis [35], show better performance in the neurocognitive function of this group [36–39] or even absence of neurocognitive differences between CU and NU with FEP [40]. One of the explanations for better neurocognitive performance is that patients who were less impaired before onset of psychosis are more prone to use cannabis [35]. The existence of moderators could explain some of the heterogeneity of previous results. Older age has been associated with better cognitive performance in verbal learning and verbal fluency in patients who use cannabis [32], and patients with a family history of psychosis performed better than patients without a family history of psychosis in verbal memory and executive function and had a higher global cognitive index [34].

This meta-analysis has the following objectives: (1) to provide a systematic review of the literature on the effects of cannabis use on neurocognitive functioning in patients with FEP; (2) to assess the effects of cannabis use on the neurocognitive functioning of FEP patients in eight domains, namely, premorbid and current intelligence quotient, attention, executive function, working memory, processing speed, verbal memory and learning, and visual memory; (3) to examine whether age at first use, duration and frequency of use, type of antipsychotic medication intake, sex, and age influenced the relationship between cannabis use and neurocognitive functioning. We hypothesized that CU with FEP would have worse neurocognitive functioning (with respect to worse attention, executive function, and verbal memory) than NU with FEP. Our second hypothesis was that a younger age at first use, a high frequency of cannabis use, and therapy with first-generation antipsychotics would be related to decreased neurocognitive functioning in CU with FEP.

## Methods

### Search strategy

The search strategy was based on the Preferred Reporting Items for Systematic Reviews and Meta-analyses (PRISMA-P) Statement [41]. Articles were identified through extensive literature searches using six online electronic databases: PubMed, ScienceDirect, Web of Knowledge, Wiley Cochrane Library, PsycInfo (EBSCOHost), and SpringerLink. The search was limited to articles in English and only peer-reviewed articles were considered. The systematic peer-review of the literature was performed independently by two researchers (T.S.-G. and S.B.). A third researcher (A.C.) was assigned for those cases in which there was no agreement in order to decide whether the manuscript met the criteria for inclusion. All of the researchers had extended expertise in psychosis. The interrater agreement was 100%. The keywords used were “first episode psychosis AND neurocognition AND cannabis,” “FEP AND cognition AND cannabis,” “Cannabis AND neurocog* AND neuropsycholog* AND FEP,” “psychosis AND cognition AND cannabis,” “FEP AND IQ AND cannabis,” “psychosis & IQ & cannabis,” and “FEP AND cognit* AND cannabis.” Weekly bibliographical alerts were created in the selected databases. These remained active until the date of submission of the present manuscript to ensure that the most recently published studies were included. Reference lists from all the studies included and from published reviews on cannabis, FEP, and neurocognition were examined.

The initial search strategy yielded 4,691 studies that were screened in the electronic databases. The abstract and methods section were read. A total of 1,640 studies were excluded because of repetition, and 2,941 were excluded because they did not fit the inclusion criteria. This resulted in a final sample of 110 studies. Further examination of the full texts of the 110 studies led to the inclusion of 7 studies in the current review, with 14 independent samples and 78 effect sizes. Supplementary Material S1 shows a flow chart of the search procedure and Table 1 shows the summarized peer-reviewed data from the studies included in the meta-analysis.

**Table 1.** Selection of studies investigating cannabis use and neurocognitive functioning

Authors	Sample, mean age (male/female)	Design	Neurocognitive measures^q^	Cannabis measures	Cannabis diagnosis	Confounding factors	Main results
Cunha et al. [36]	28 FEP^a^ CU^f^ Age: 24.1 ± 6.5 (18/50) 78 FEP NU^g^ Age: 29.3 ± 8.6 (18/50) 80 HC^b^ Age: 30.4 ± 8.3 (18/50)	Cross-sectional	Attention: WAIS-III^d^ digits forward subtest Working memory: letter-number sequencing Verbal fluency and executive functioning: FAS^h^ and COWAT^i^	Age of first use, duration (years), frequency (daily, 2–3 times/week, 1–3 times/month), current cannabis use, previous cannabis use	Use, abuse, and dependence	Age and sex	FEP CNU presented cognitive impairments in attention compared with HC and cognitive impairments in working memory compared with FEP CU and HC
Leeson et al. [37]	34 FEP NU Age: 28.29 ± 10.87 (16/60) 65 FEP NU Age: 23.42 ± 6.06 (16/60)	Longitudinal	Verbal learning: Rey auditory verbal learning task Working memory manipulation: CANTAB^j^ Working memory span: CANTAB Planning: Tower of London task (total number of perfect solutions) Premorbid IQ^c^: WTAR^e^ Current IQ: WAIS-III subtests: information, block design, arithmetic, and digit symbol	Frequency, years between first cannabis use and FEP onset, age of first use	Use	IQ	Cannabis use may bring forward the onset of psychosis in people who otherwise have good prognostic features, as indicated by premorbid Cognition and social function At the onset of psychosis, cannabis users exhibited better cognitive function than never-users and there were no differential group, changes in cognition over the following 15 months The group differences in every subtest vanished after the introduction of premorbid IQ as a control variable, thus reflecting higher intellectual functioning in the cannabis users prior to onset of psychosis
Rodríguez-Sánchez et al. [39]	47 FEP CU Age: 23.62 ± 4.15 (15/60) 57 FEP NU Age: 30.04 ± 8.47 (15/60) 37 HC Age: 25.24 ± 7.87 (15/60)	Longitudinal	Verbal memory: Rey auditory verbal learning test Visual memory: Rey complex figure test Motor speed: tapping test Executive function: TMT-B^k^ Working memory: WAIS-III backward digits Speed of processing: WAIS-III digit symbol Verbal fluency: FAS Attention: number of correct responses of CPT^l^ Motor coordination: Grooved Pegboard (seconds to complete the board with the dominant hand as the dependent variable) Premorbid IQ: WAIS-III vocabulary subtest	Frequency (within the previous year), duration (at program entry), and age of first use	Use	Age, years of education, premorbid IQ, and baseline performance in follow-up analysis	Cannabis users with schizophrenia have better attention and executive functions than nonusers at baseline and after 1 year of treatment Both groups of patients showed a similar increase in their performance in cognitive tasks during the 1-year follow-up period The amount of cannabis consumed and the length of time of consumption did not significantly influence cognitive performance
Yücel et al. [38]	59 FEP CU Age: 20.7 ± 2.8 26 FEP NU Age: 20.6 ± 3.5 43 HC59 FEP CU Age: 21.6 ± 5.8	Cross-sectional	Premorbid IQ: National adult reading test Current IQ: WAIS-R Processing speed: TMT-A^m^ and digit symbol coding Verbal memory: Rey auditory verbal learning test and the logical memory component of the WMS-R^n^ Visual memory: visual paired associates (part I) and Visual Reproduction (part I) from the WMS-R; spatial and pattern recognition tests from the CANTAB Working memory: spatial span and spatial working memory from the CANTAB Executive functioning (planning and reasoning): block design from the WAIS-R and the Tower of London	Age of first use, frequency, recency	Use, abuse, and dependence	Sex and age	Comorbid cannabis use is associated with a superior cognitive profile in schizophrenia spectrum disorders FEP patients who used cannabis (especially those who used cannabis prior to age 17) performed better than nonusing patients and were less impaired than healthy controls in visual memory, working memory, planning, and reasoning
de la Serna et al. [42]	32 FEP CU Age: 16.34 ± 0.16 (9/17) 76 FEP NU Age: 15.16 ± 0.22 (9/17) 96 HC Age: 15.18 ± 0.19 (9/17)	Cross-sectional	Attention: WAIS-III digits forward, TMT-A, Stroop test (words + colors), CPT (number of correct responses and average time reaction) Working memory: WAIS-III digits backward, WAIS-III number-letter sequencing Learning and memory: TAVEC^o^ Executive functions: TMT-B, FAS and COWAT, Stroop test (interference), WCST^p^	Presence or absence of cannabis use in the previous month	Use	Age, socioeconomic status	Significant differences in overall cognitive functioning in controls compared with both groups of patients (CU and NU) Better cognitive functioning in the CU group compared with the NU group on tasks assessing attention and executive functions
Mata et al. [33]	61 FEP CU Age: 23.42 ± 4.14 (9/17) 71 FEP NU Age: 29.54 ± 8.44 (9/17)	Cross-sectional	Premorbid IQ: verbal comprehension index (WAIS-III) Decision-making: Iowa gambling task Working memory: WAIS-III digits backward Executive function: FAS and TMT-A and TMT-B	Presence or absence of cannabis use in the previous year	Use, abuse, and dependence	Age and sex, previous use of other drugs	Cannabis abuse prior to the onset of psychosis is associated with greater impairment in a decision-making task linked to orbitofrontal function but not with more severe deficits in the performance of working memory and executive function tasks, which are sensitive to dorsolateral prefrontal function Patients who abused cannabis also had lower estimated IQ than nonusers
Moreno Granados et al. (2014)	41 FEP Age: 16.15 ± 2.6 (12–18) 39 HC Age: 17.38 ± 2.37 (12–18)	Cross-sectional	Verbal memory: WMS-III Visual memory: Rey complex figure test	Frequency of lifetime use, age at first use, current use, lifetime exposure, severity	Use, abuse, and dependence	Age and sex, previous use of other drugs	A significant interaction of time by group was observed in the processing speed domain, only in the male subgroup. However, among patients with schizophrenia, cannabis users performed better than nonusers

a FEP: first-episode psychosis.

b HC: healthy controls.

c IQ: intelligence quotient.

d WAIS-III: Wechsler Adult Intelligence Scale, 3rd edition.

e WTAR: Wechsler test of adult reading.

f CU: cannabis users.

g NU: nonusers.

h FAS: verbal fluency test.

i COWAT: Controlled Oral Word Association.

j CANTAB: Cambridge Automated Neuropsychological Test Automated Battery.

k TMT-B: trail making test, part B.

l CPT: continuous performance test.

m TMT-A: trail making test, part A.

n WMS-R: Wechsler Memory Scale-Revised.

o TAVEC: Test de Aprendizaje Verbal España-Complutense (Spanish version of the California verbal learning test.

p WCST: Wisconsin Card Sorting Test Index.

q Authors of each study did not provide a global or independent score for each of the domains that were measured. See Table 2 for the correspondence between the subtest and the neuropsychological domains.

### Selection criteria and data extraction

Cross-sectional and longitudinal studies were included in the systematic review when they met the following criteria: (1) diagnosis of FEP according to the Diagnostic and Statistical Manual of Mental Disorders (patients with psychotic symptoms who could have received antipsychotic treatment for less than 12 weeks); (2) comparison between CU with FEP and NU with FEP; (3) cannabis abuse or dependence with no other comorbid substance use disorder (except for the common mixture of tobacco and cannabis in the same cigarette when patients did not report independent tobacco use); (4) assessment of neuropsychological functioning based on valid and reliable tests commonly used in clinical practice; and (5) sufficient statistical data for transformation into effect sizes from the original researchers. The reasons for exclusion were as follows: (1) diagnosis of a category other than FEP within the psychosis spectrum (e.g., schizophrenia, substance-induced psychotic disorders, schizoaffective disorders); (2) studies on the effects of individual components of cannabis on cognitive functioning; (3) studies in which participants had poly-substance use disorders, even if there was a preferential use toward cannabis, given that other substances of abuse (e.g., alcohol, cocaine, and stimulants) are associated with altered cognitive performance [42,43]; (4) studies whose main neuropsychological outcomes required MRI-based assessment; (5) available data on cannabis use classified according to more than two different levels of use (e.g., NU plus 2 or more cannabis use pathways).

The search was limited to studies published during the last 10 years (2008 to July 2018). The studies included were coded according to the first author using a coding sheet. The outcome variable was coded as “neurocognitive domains.” In some cases, the neuropsychological instruments used to measure the cognitive domains were different. Therefore, we grouped the subtest into the following domains: current intelligence quotient (IQ), premorbid IQ, executive function, attention, working memory, verbal memory and learning, visual memory, and processing speed. Details on the correspondence between subtests and neuropsychological domains can be seen in Supplementary Material S2.

Other variables were coded according to whether they applied to CU with FEP or NU with FEP. The moderator variables collected in the CU group were mean age at first use, mean duration of lifetime cannabis use in years, and frequency of cannabis use, distinguishing between low frequency (two times or less per week) and high frequency (three times or more per week). Moreover, we extracted the type of antipsychotic medication (coded as first-generation or second-generation antipsychotics) and sociodemographic data such as sex, age, and country where the study was performed. Interrater agreement for the calculated effect was 100%.

### Calculations and analyses

From each study, the information needed to calculate standardized mean differences (Cohen’s *d*) and their sampling variances was extracted. This information consisted of the means, SDs, and sample sizes of the two independent groups (CU and NU). Standardized mean differences were then corrected for bias by transforming them to Hedges’ *g* [44]. A positive Hedges’ *g* indicates that NU score higher in cognitive tests than CU.

All studies reported more than one effect size, because the independent groups were compared in several domains of neurocognitive performance (see Supplementary Material S2). Since effect sizes extracted from the same sample are related to each other, the application of classic meta-analytic techniques would violate the assumption of independent effect sizes. Violating this assumption, that is, ignoring dependency among effect sizes, can lead to a biased estimate of the standard error of the pooled effect and biased variance component estimates [45]. Therefore, in order to account for dependent effect sizes, a three-level model was applied [46,47]. With the application of a three-level model, it is possible to model three different sources of variance: the sampling variance observed at Level 1 (which is known and calculated beforehand), the variance between the effect sizes belonging to the same study at Level 2 (within-study variance), and the variance between study effects at Level 3 (between-studies variance).

Log-likelihood-ratio-tests were performed to determine whether the within-study (Level 2) and between-studies (Level 3) variances were significant [48]. These tests compare the deviance of the full model (three-level model) with the deviance of the model where the between-studies variance has been ignored and with the deviance of a model that does not take into account the within-study variance. If the results of these two tests are statistically significant, then it can be concluded that there is significant variance at both levels. Furthermore, the percentage of variability due to systematic differences between studies, due to systematic differences within studies, and due to sampling variance was calculated using the formulae of Cheung [49]. In order to explain these variances, moderator variables were introduced in the model one by one. Continuous moderator variables (i.e., age at first use, duration of use, percentage of low-frequency users, percentage of high-frequency users, percentage of participants taking first-generation antipsychotics, percentage of participants taking second-generation antipsychotics, percentage of men, and age) were centered to their mean for ease of interpretation. For some moderator variables (i.e., age, percentage of men, percentage of participants taking first-generation antipsychotics, and percentage of participants taking second-generation antipsychotics), the information was available for both the user group and the nonuser group. Therefore, we first performed an independent *t* test, and if no differences were found between CU and NU, the values of the moderator variables were averaged across the groups. However, if differences between the groups emerged, we performed a two-step analysis in which the data from moderators of the CU group were first introduced in the model, followed by the moderator referring to the NU group.

The validity of the results of the meta-analysis was threatened by possible publication bias. That is, if studies reporting nonsignificant results were censored or had never been submitted for publication, our sample of studies could be biased, as could, therefore, our results [50]. In this meta-analysis, the presence of publication bias was evaluated by visually inspecting the funnel plot [51] and by applying a three-level version of the Egger regression test [52]. We also performed sensitivity analyses for outliers by excluding, one by one, effect sizes that were larger or smaller than two SDs above/below the mean.

All analyses were performed with the PROC MIXED procedure in SAS, which uses restricted maximum likelihood as the estimation method. Funnel plots were generated with R.

## Results

### Final sample of studies

The meta-analysis of the effect of cannabis use on neurocognitive performance in patients with FEP contained 7 independent studies (*k*) reporting on 78 effect sizes (*m*) and a total sample of 673 subjects. The sample consisted of 304 subjects in the CU group and 369 subjects in the NU group.

### Overall analyses

The three-level meta-analysis resulted in a nonsignificant overall effect of −0.05 (SE = 0.15, *p* = 0.96, 95% confidence interval [CI; −0.39, 0.37]). The log-likelihood-ratio tests revealed that the between-studies variance was not statistically significant (*χ^2^* = 2.30, *df* = 1, *p* = 0.13), while the within-study variance was statistically significant (*χ^2^* = 640.2, *df* = 1, *p* < 0.001). Around 8.5% of the total variance in effect sizes was due to differences between studies, 86.7% of the total variance between effect sizes was attributed to differences within studies, and 4.8% of the observed variability was due to sampling variance.

### Independent analysis of neurocognitive domains

**Figure 1.**
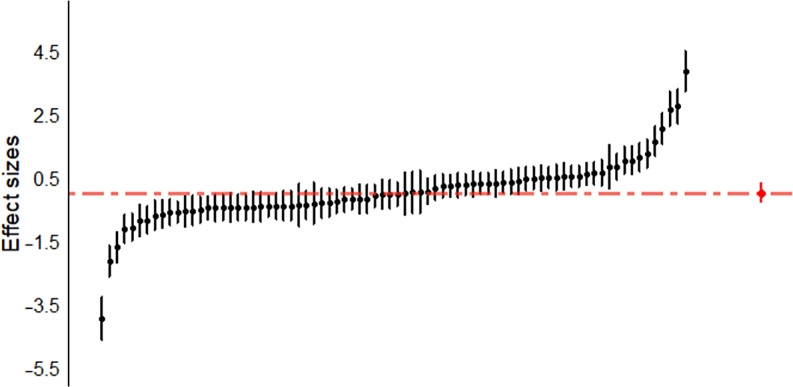
Caterpillar plot. Individual effect sizes (*n* = 78) of all of the cognitive domains (attention, executive function, processing speed, verbal memory and learning, visual memory, working memory, and premorbid IQ) are represented.

Nonsignificant effects were observed in the analyses of each of the cognitive domains separately: (1) attention (*d* = −0.35, *p* = 0.68); (2) executive function (*d* = 0.16, *p* = 0.59); (3) premorbid IQ (*d* = 0.10, *p* = 0.72); (4) processing speed (*d* = 0.15, *p* = 0.69); (5) verbal memory and learning (*d* = −0.005, *p* = 0.98); (6) visual memory (*d* = −0.08, *p* = 0.78); and (7) working memory (*d* = −0.04, *p* = 0.90; see Table 2). Figure 1 shows the caterpillar plot where all the effect sizes are represented.

**Table 2.** Analyses of the effects of cannabis use on the neuropsychological performance domains

Outcome variables^a^	*k* ^c^	*m* ^d^	*n* ^e^ CU^f^	*n* CNU^g^	*β* ^h^ (SE)^i^	95% CI^j^	*t* (*df*)^k^	*p* ^l^
Overall	7	78	304	369	−0.009 (0.155)	(−0.389, 0.372)	−0.06 (6.54)	0.957
Attention	3	8	107	211	−0.350 (0.806)	(−2.262, 1.561)	−0.43 (6.90)	0.677
Executive function	6	28	292	342	0.165 (0.283)	(−0.618, 0.949)	0.58 (4.06)	0.591
Premorbid IQ^b^	4	4	232	188	0.097 (0.248)	(−0.692, 0.886)	0.39 (3)	0.721
Processing speed	2	3	106	83	0.150 (0.280)	(−3.408, 3.708)	0.53 (1)	0.687
Verbal memory and learning	5	14	215	220	−0.005 (0.183)	(−0.400, 0.389)	−0.03 (13)	0.977
Visual memory	3	7	118	110	−0.076 (0.244)	(−1.138, 0.984)	−0.31 (1.98)	0.783
Working memory	6	10	292	342	−0.037 (0.295)	(−0.800, 0.725)	−0.13 (4.92)	0.904

a For motor coordination and current IQ, there were not enough observations to perform the analyses.

b For premorbid IQ, a standard two-level random effects model was applied because there was only one outcome per study.

c *k*: number of studies that report information about the moderator variable.

d *m*: number of effect sizes that report information about the moderator variable.

e *n*: sample size.

f CU: cannabis user group.

g CNU: cannabis nonuser group.

h *β*: mean effect size.

i SE: standard error.

j CI: confidence interval.

k *df*: degrees of freedom.

l *p*: significant *p* value (*p* ≤ 0.005).

### Moderator analyses

The independent *t* tests revealed no differences between CU and NU regarding the percentage of participants taking first- and second-generation antipsychotic medication (*t* = 0.314, *df* = 6, *p* = 0.37 for both analyses). Similarly, no differences were found in the mean age (*t* = 0.233, *df* = 10, *p* = 0.23), although there were differences across groups regarding the percentage of men (*t* = 5.307, *df* = 10, *p* < 0.001). This percentage was higher in CU (mean = 75.35, SD = 5.77) than in NU (mean = 52.39, SD = 8.88). Therefore, we present the effect of the percentage of men in CU and the percentage of men in NU separately.

When analyzing the effects of the moderator variables on cannabis use and neurocognitive functioning, we found that a low frequency of use led to a more positive effect size, that is, the difference in the neurocognitive functioning between CU and NU was greater, in favor of NU (*F* = 7.56, *df* = 1, 12.5, *p* = 0.02). Moreover, the use of first-generation antipsychotics led to a greater difference in neurocognitive functioning between the groups (*F* = 34.46, *df* = 1, 47, *p* < 0.001). Finally, the country where the study took place influenced the neurocognitive functioning of both groups (*F* = 3.75, *df* = 3, 70.5, *p* = 0.01, see Table 3).

**Table 3.** Moderator effect of selected variables on cannabis use and neurocognitive functioning

Moderator variable	*k* ^a^	*n* ^b^	*β* ^c^	*F* ^d^	*df* ^e^	*p* ^f^
Duration	4	36	0.038	0.05	1, 1.88	0.851
Age of first use	4	36	0.649	3.41	1, 2.05	0.203
Low frequency CU	3	15	0.013	7.56	1, 12.5	**0.017**
High frequency CU	3	15	−0.041	2.37	1, 1	0.366
Medication FG^g^	4	49	0.019	34.46	1, 47	**<0.001**
CU% men^h^	6	72	0.006	0.03	1, 3.16	0.881
CNU% men^i^	6	72	0.026	1.27	1, 4.42	0.317
% men	7	78	0.038	1.17	1, 8.73	0.309
Age	7	78	−0.029	0.72	1, 4.9	0.437
Country				3.75	3, 70.5	**0.015**
United Kingdom	1	6	−0.353	*t =* −0.92	68.0	0.358
Brazil	1	3	−0.760	*t =* −1.40	68.9	0.166
Spain	4	53	0.266	*t =* 2.06	68.9	**0.043**
Australia	1	16	−0.403	*t =* −1.71	69.6	0.091

a *k*: number of studies that report information about the moderator variable.

b *n*: number of effect sizes that report information about the moderator variable.

c *β*: mean effect size.

d *F*: Value of the statistic Snedecor’s F

e *df*: degrees of freedom.

f *p*: significant *p* value (*p* ≤ 0.005).

g FG: first generation antipsychotics.

h CU: cannabis users.

i CNU: cannabis nonunsers. Probability values in bold represent significant results (*p* ≤ 0.05).

### Sensitivity analyses

Five outliers were detected (three positive and two negative), all in the study of de la Serna et al. [53]. By deleting each of these outliers one by one and performing the analyses again each time, the overall effect ranged from −0.004 (95% CI [−0.44, 0.433], *p* = 0.98) to −0.04 (95% CI [−0.37, 0.29], *p* = 0.84). These results indicate that the overall estimate is quite robust.

### Publication bias

**Figure 2.**
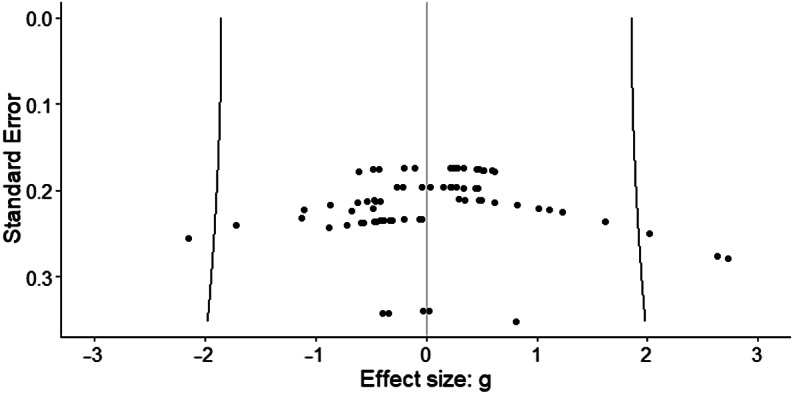
Funnel plot. The fact that effect sizes are symmetrically distributed around the mean effect seems to indicate the absence of publication bias.

Figure 2 shows the funnel plot with all of the effect sizes. The fact that effect sizes are symmetrically distributed around the mean effect seems to indicate the absence of publication bias. Furthermore, the three-level Egger regression test showed no significant association between the standard errors and their corresponding effect sizes (*F* = 0.01, *df* = 1, 14.64, *p* = 0.97).

## Discussion

The results of this three-level model meta-analysis revealed no significant differences between CU and NU with respect to neurocognitive functioning. These findings are consistent with those of some previous studies, which show an absence of differences in neurocognitive functioning between CU and NU with FEP [40], but not with those of others [32]. The lack of significant differences in neurocognitive functioning between CU and NU with FEP in this study could be explained by the fact that vulnerable patients with better cognitive function can develop psychosis owing to cannabis use; this subsample can mask the deleterious effect of cannabis on cognition in other patient groups. In addition, the results can be explained by the different times of exposure to cannabis. Cumulative exposure to cannabis in a developing vulnerable adolescent brain accounts for an increased risk of onset of psychosis and, therefore, the manifestation of its symptoms [16-18], including the effects of the disease itself on neurocognitive functioning [32]. The present results may have been masked by the different durations of cannabis use. However, most of these results have to be interpreted with caution owing to the small number of observations (i.e., processing speed and current IQ), thus revealing the lack of sufficient power to detect a significant effect. Future research might investigate the effect of duration of cannabis exposure prior to the onset of FEP and test whether longer exposures translate into effects on neurocognitive functioning.

The analysis of the moderator variables showed that the percentage of participants who less frequently used cannabis had a more positive influence on the pooled effect, that is, the higher the number of participants who used cannabis less frequently, the better the performance of the NU in cognitive domains (and the worse the performance of users). This remarkable and counterintuitive result can be explained by differences in the underlying mechanism of psychosis associated with frequency of use for CU. Heavy, repeated cannabis use, particularly during adolescence in patients without psychosis, has been associated with adverse effects on the endocannabinoid system, especially in genetically predisposed persons [54]. Chronic and heavy use of cannabis in persons without psychosis affects cognitive domains such as memory and attention, as well as their associated brain areas [55]. However, heavy use in patients with a very high genetic risk (family history of psychosis) is associated with better results in some cognitive domains, such as verbal learning [29]. As for lower use of cannabis and psychosis, a recent study found a cluster association between poor cognition and low cannabis use in comparison with moderate and heavy use [56]. The endocannabinoid system restores homeostasis in brain functioning during brain damage, such as the neurocognitive impairment that is observed in patients with psychosis [23]. The presence of exogenous cannabis is one of the main factors accounting for the dysregulation of the endocannabinoid system [26]. As a result, the use of cannabis, even at low doses, in adolescents with a low threshold for developing psychosis after cannabis use may trigger the dysregulation of this system and accelerate the incidence of positive, negative, affective, and cognitive psychotic symptoms [16-18,32]. Consequently, the results of the present meta-analysis may support the finding that cannabis use acts as a risk factor for the worsening of cognitive function in a subsample of patients with a low threshold for developing FEP in CU [33,36,37]. Further analysis must investigate whether cognition in this subsample improves once cannabis is no longer used.

The analysis revealed significant effects of first-generation antipsychotics and second-generation antipsychotics. Within the group of patients taking first-generation antipsychotics, the percentage of drugs increased with differences in neurocognitive functioning between CU and NU with FEP. Thus, CU scored higher in all of the neurocognitive domains despite the absence of significant results. These findings are consistent with studies on the differential antidopaminergic effects of first and second-generation antipsychotics and the disturbing effects of cannabis use on the homeostatic effects of endocannabinoids such as anandamide and 2-arachidonoylglycerol (2-AG) [57,58]. FEP patients present increased endocannabinoid levels in the frontal cortex and in cerebrospinal fluid even before using cannabis [24,25]. This was mainly due to the counteracting effects of anandamide and 2-AG in patients with psychosis and in individuals at risk for psychosis who present increased levels of dopamine in the mesolimbic areas [59-61]. The endocannabinoid system increases the level of anandamide and 2-AG to naturally balance dopamine hyperactivation in mesolimbic areas [62]. However, first-generation antipsychotics antagonize the postsynaptic dopaminergic D2 receptors, thus decreasing levels of dopamine [63] and helping endocannabinoids to achieve homeostasis. This pharmacological action implies an excessive decrease in dopamine in the mesocortical circuits, thus increasing the onset of negative and cognitive symptoms. In contrast, second-generation antipsychotics interact not only with the dopamine system, but also with the serotoninergic neurotransmitter system [64], thus decreasing the incidence of negative and cognitive symptoms of psychosis [57]. Cannabis use may dysregulate both the homeostasis task of the endocannabinoid system [65,66] and worsen the pharmacological effects of antipsychotics. This result may have clinical implications for the development of patient-focused medicine, which implies the selection of second-generation antipsychotics in CU.

Our study also reveals a significant country effect. It seems that studies performed in Spain yielded a higher (positive) effect size, which was significantly different from zero, meaning that NU score higher than CU in cognitive domains. In contrast, no significant effects were found in the other countries. These results should be interpreted with caution because most of the studies that met the criteria for the present meta-analysis were carried out in Spain. Consequently, Spain is overrepresented in our data. Even so, this outcome might be explained by the differences in cannabis preparations worldwide and the use of genetically modified plants with a high THC content, such as the popular skunk or *sinsemilla*, which is used predominantly in Central Europe and the UK [4,5,7]. These cannabis products have significantly decreased CBD [67], one of the several cannabinoids present in the *Cannabis sativa* plants. CBD has shown antipsychotic properties and preserves cognitive deterioration effects in patients with psychosis [68-70]. In conclusion, the dose, frequency, and different types of cannabis preparations may interfere with the neurocognitive functioning of CU with FEP.

The results of this study show the importance of some moderators in the cognitive skills of patients with psychosis. However, as these results are associations, we cannot infer causal relationships. In addition, heavy cannabis use in patients with chronic schizophrenia leads to worse cognitive performance than in moderate users [56], and this can be explained by the deleterious chronic effect of cannabis use in the long term, a finding not reported in the FEP samples studied in our meta-analysis. In addition, it is important to remember that quitting cannabis improves functionality in FEP in the long term [29,34].

The strengths of the present study include the focus on a large sample of FEP patients and specific neurocognitive domains and the inclusion of studies in which patients only used cannabis. The study takes into account the shortcomings of previous systematic reviews and meta-analyses. Furthermore, the multilevel approach made it possible to include moderator variables. Therefore, we were able to assess the influence of cannabis duration and frequency of use, as well as demographic and clinical variables, on neurocognitive functioning outcomes [46,47], thus gaining more insight into interference with the neurocognitive performance of FEP patients. Our study is also subject to a series of limitations. First, the relatively small number of effect sizes in some of the neurocognitive domains analyzed could mean that the statistical power is insufficient to detect a significant effect between the groups. Second, some moderator variables, such as the country where the study was developed, were very uneven in terms of observations within categories. Other moderator variables such as, the educational level, which was described as a potential variable that could influence cognitive functioning [71] and antipsychotic dosage were not included in this study. Only the study of Yücel et al. [38] comprised doses of chlorpromazine equivalents for each comparison group. Future studies in this field could consider the selection of these moderator variables to analyze its relationship with cognitive functioning in cannabis-users. Third, the consideration of antipsychotic treatment for the definition of FEP was not a homogenous criterion in each of the studies. While the studies of Cunha et al. [36], Yücel et al. [38], de la Serna et al. [53], and Moreno-Granados et al. [72] did not include information regarding previous antipsychotic treatment to the enrollment in the study, the studies of Leeson et al. [37], Rodríguez-Sánchez et al. [39], and Mata et al. [33] enrolled FEP patients who could have received neuroleptic medication for more than 12, 6, or 4 weeks, respectively. Future research may account for this limitation in order to observe if there were any cofounding effects regarding antipsychotic medication exposition at the onset of the disorder. Fourth, due to the wide exclusion criteria selected for this study, it could be considered that the sample obtained for this meta-analysis is not naturalistic. Finally, all of the studies included in the meta-analysis were cross-sectional; therefore, a longitudinal follow-up of these studies could help elucidate whether the absence of differences, the duration of psychosis, and the level of cumulative cannabis exposure all remain the same.

## Conclusions

In conclusion, the current study shows that cannabis use is not related to the neurocognitive functioning of patients with FEP. It also highlights the importance of moderators. Future research is needed to understand how the neurocognitive functioning of patients with FEP is affected by dose, duration of cannabis exposure, and different types of cannabis preparations, as well as the potential interactions between antipsychotic medication and cannabis use.

## Supplementary material

For supplementary material accompanying this paper visit https://doi.org/10.1192/j.eurpsy.2019.9. click here to view supplementary material
