# Supplementary material for: Cannabis use and nonuse in patients with first-episode psychosis: A systematic review and meta-analysis of studies comparing neurocognitive functioning
Source: Eur Psychiatry. 2020 Jan 31;63(1):e6. doi: 10.1192/j.eurpsy.2019.9 (PMC8057396; doi:10.1192/j.eurpsy.2019.9)
Supplement: Supplementary file 1 [file epasup.zip › S0924933819000099sup002.docx]

**Supplementary material 2. Correspondence between the neuropsychological tests and the cognitive domains used in the meta-analysis**

| **Cognitive domain** | **Neuropsychological tests** |
| --- | --- |
| **Current IQ^a^** | WAIS-III^b^ (Information, Digit Span, Matrix, Block Design, and Arithmetic) and WAIS-R^c^ |
| **Premorbid IQ** | WTAR^d^, WAIS-III (Vocabulary, Information, and Similarities subtests) and National Adult Reading Test |
| **Executive function** | COWAT^e^ and FAS^f^ (number of words and semantic categories), Tower of London task (total number of perfect solutions), TMT-B^g^, Block design, Stroop interference, WCST^h^ and Decision-making (Gambling task) |
| **Attention** | Digits Forward (WMS-III^i^), Digits Forward (WAIS-III), CPT^j^, Time to complete TMT-A^k^, and No. of correct items in Stroop words and colours |
| **Working memory** | Backward Digits (WMS-III), Digits Backward and number-letter sequencing (WAIS-III), CANTAB^l^, Spatial Working Memory-errors, and strategy and Spatial Span |
| **Verbal memory and learning** | Rey Auditory Verbal Learning Task (List A total trials and long-term recall), Logical Memory, TAVEC^m^, WMS-R, and WMS-III |
| **Visual memory** | Rey Complex Figure, Visual Paired Associates (part I) and Visual Reproduction (part I) (WMS-R^n^), Spatial Recognition and Pattern Recognition (CANTAB) |
| **Processing speed** | Digit Symbol WAIS-III, TMT-A |

^a^IQ: intelligence quotient; ^b^WAIS-III: Wechsler Adult Intelligence Scale, 3^rd^ edition; ^c^WAIS-R: Wechsler Adult Intelligence Scale-Revised ^d^WTAR: Wechsler Test of Adult Reading; ^e^COWAT: Controlled Oral Word Association; ^f^FAS: Verbal Fluency test; ^g^TMT-B: Trail Making Test, part B; ^h^WCST: Wisconsin Card Sorting Test Index; ^i^WMS-III: Wechsler Memory Scale-3^rd^ Edition; ^j^CPT: Continuous Performance Test; ^k^TMT-A: Trail Making Test, Part A; ^l^CANTAB: Cambridge Automated Neuropsychological Test Automated Battery; ^m^TAVEC: Test de Aprendizaje Verbal España-Complutense (Spanish version of the California Verbal Learning Test; ^n^WMS-R: Wechsler Memory Scale-Revised
