# Supplementary figures and images for: Cannabis use and nonuse in patients with first-episode psychosis: A systematic review and meta-analysis of studies comparing neurocognitive functioning
Source: Eur Psychiatry. 2020 Jan 31;63(1):e6. doi: 10.1192/j.eurpsy.2019.9 (PMC8057396; doi:10.1192/j.eurpsy.2019.9)

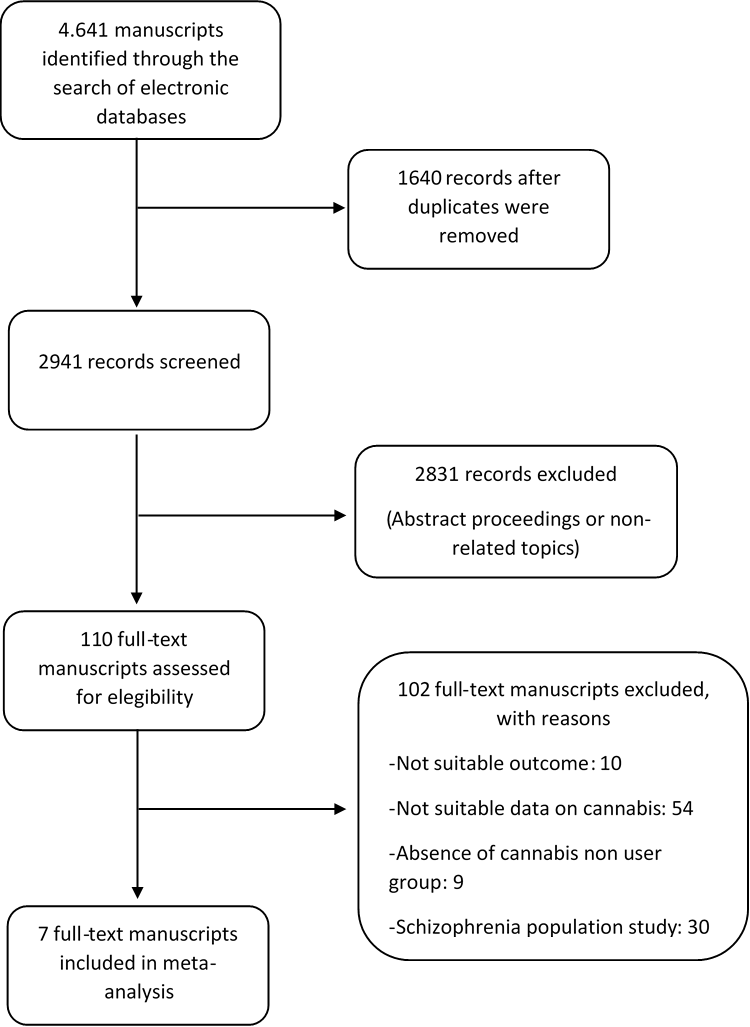


**Supplementary material 1: PRISMA flowchart of the study selection process**

Supplement: Supplementary file 1 [file epasup.zip › S0924933819000099sup001.docx]
